# Knowledge, Perceptions and Attitudes Toward HPV Vaccination: A Survey on Parents of Girls Aged 11–18 Years Old in Greece

**DOI:** 10.3389/fgwh.2022.871090

**Published:** 2022-06-16

**Authors:** Panagiota Naoum, Kostas Athanasakis, Dimitris Zavras, John Kyriopoulos, Elpida Pavi

**Affiliations:** Laboratory for Health Technology Assessment (LabHTA), Department of Public Health Policy, School of Public Health, University of West Attica, Athens, Greece

**Keywords:** HPV, human papillomavirus, adolescent vaccination, parent perceptions, health education

## Abstract

**Objective:**

To investigate knowledge, perceptions and practices of parents of girls aged 11–18 years old in Greece toward HPV vaccination, and determine which factors are associated with parents' decision to vaccinate their daughters.

**Methods:**

A close-end questionnaire was constructed and telephone interviews were conducted upon informed consent. The sample was random, national, stratified by geographic region and representative of the general population of parents of girls aged 11–18. The data collected include: general knowledge, attitudes and perceptions concerning HPV and HPV vaccine, information regarding their daughters' HPV vaccination, and sociodemographic characteristics. Statistical analysis included descriptives and a logistic regression model to investigate which factors are associated with HPV vaccination.

**Results:**

Overall, 1,000 parents participated in the study, 99.4% of which knew what HPV is and 98.8% knew there is a vaccine available against HPV. Furthermore, 47% of the parents stated that their daughters had been vaccinated against HPV, while further analysis revealed that only 35% had received all the recommended doses. In the logistic regression analysis, the following variables had a statistically significant association with HPV vaccination: perceived ease of contracting HPV (OR = 1.105), level of trust in medical profession regarding information on prevention (OR = 1.205), overall perception regarding importance of children's vaccination (OR = 0.618), internet/social media as a source of parent information regarding HPV (OR = 0.886), participant (parent) age (OR = 1.125), and daughter's treating physician's recommendation for HPV vaccination (OR = 7.319).

**Conclusions:**

HPV vaccination coverage is still suboptimal. Comprehension of the obstacles toward this goal is important and the role of healthcare professionals is crucial to increase acceptance.

## Introduction

Human Papilloma Virus (HPV) is the main cause of almost every case of cervical cancer, while it is also responsible for a number of anogenital and oropharyngeal cancers (1). The main mode of HPV transmission is sexual contact, but research has shown that it can be also transmitted in different ways, such as transmission from mother to child during childbirth (2).

Cervical cancer is the fourth most common cancer type in women worldwide, accounting for 604,127 cases in 2020, while it is also the fourth cause of cancer death in women globally with 341,831 deaths (3). In Greece, cervical cancer is the tenth most common type of cancer and the twelfth cause of cancer death, based on 2020 data (4).

HPV vaccination is considered as one of the most effective practices against HPV (5, 6), while a variety of data confirm its safety profile (7, 8). The international literature has demonstrated that HPV vaccination programmes can reduce the burden of disease for diseases associated with HPV (9), such as cervical cancer, and their respective costs (10).

Greece, in line with various other countries, has added HPV vaccination in its National Programme of Vaccination for children and adolescents (11). In particular, HPV vaccination (bivalent HPV for two or 9vHPV for nine subtypes, respectively) is offered with no copayment for girls aged 11–18 years old, as a 2-dose scheme for those aged 11–14 years old and as a 3-dose scheme for those aged 15–18 years old. It is also offered with no copayment for boys aged 11–18 years old which belong to high risk groups of the population, such as those who are immunosuppressed.

As reported (12), in order to achieve optimum cost-effectiveness of HPV vaccination, more than 70% of the female population must be vaccinated. However, free of charge availability of the vaccine has proved to be inadequate to achieve high population coverage (13). Low rates of vaccination uptake can be attributed to a variety of factors, such as socioeconomic, cultural, and religious determinants, as well as inequity in access (14–16). Based on the fact that HPV vaccination is recommended for children and adolescents, acceptance by parents is the main driver toward increase in HPV vaccination coverage rates.

Based on the above, aim of the present study was to investigate various aspects of knowledge, perceptions and practices of the Greek population, and more particularly parents of girls aged 11–18 years old, toward HPV vaccination, as well as to determine which factors are associated with parents' decision to vaccinate their daughters.

## Materials and Methods

In order to obtain the necessary data for the analysis, a purpose made close-end questionnaire was constructed. The sample was random, stratified by geographic region and urbanization level, was national and representative of the general population of parents of girls aged 11–18 in Greece and was drawn as follows:

Geographic stratification/urbanization for the Greek adult population (target population) was employed in order to ensure proportional representation for the national sample. Computer Assisted Telephone Interviews (CATI) system automatically generated random telephone numbers. For reasons of anonymity, the professional and specially trained interviewers did not know the telephone number of the interviewee. Upon informed consent, telephone interviews were carried out in morning, afternoon, and evening, to ensure that both working and not working parents were reached.

Filter questions for the fulfillment of the inclusion criteria were: (a) region of residence and urbanization level, (b) having children, (c) gender and age of all children, (d) if 1+ girls aged 11–18 years, inclusion in the study, (e) if 2+ girls aged 11–18 years, selection of one girl based on a random digits table, and information given to parent participant that for the interview the daughter selected should be kept in mind, (f) that the responder is the parent who participates in the decision-making for health issues of the daughter, and (g) knowledge of the HPV vaccine. Study interviews were conducted during June-July 2019 and all necessary ethics approvals were obtained from the Bioethics and Research Ethics Committee of the Hellenic National School of Public Health (3568/9.11.2018).

For the questionnaire construction, a thorough review of the existing literature on the subject was performed by the research team, in order to acquire the necessary items regarding the factors/determinants that are associated with the decision toward vaccination against HPV. Accordingly, the acquired items were transformed into questions, based on the general context of the KAP method (Knowledge, Attitudes, and Practices).

The questionnaire of the study consisted of the following broad question categories: (a) general knowledge concerning HPV as a virus and its vaccine, (b) attitudes and perceptions toward the vaccine, (c) whether their daughter has been vaccinated against HPV and why/why not. Additional data were collected on various sociodemographic characteristics of the participants. Also, it should be noted that, while most questions were either categorical or had specific levels of scales, a few of the items in the questionnaire required the participants to respond in a numerical form on a 0–10 scale (0: not at all; 10: extremely). In particular, these were: degree of avocation with religion, trust in various sources to inform the public on prevention and how easy people believe it is to get HPV.

Additionally to the main reporting whether the respondents' daughters had been vaccinated against HPV or not, the status of vaccination (full or not) was also estimated. In particular, this estimation was based on the age that the girls received the first dose of the HPV vaccine and the respective doses required for full vaccination, according to the National Programme of Vaccination for children and adolescents (11).

The statistical analysis was performed with the SPSS statistical software (v.20). Additionally to the descriptive analysis of the sample, a binary logistic regression analysis was also employed, in order to investigate which factors are associated with the decision to vaccinate. In this analysis, the dependent variable was the status of vaccination, while the independent variables were selected on the basis of the univariate associations of the various items of the questionnaire with vaccination coverage, as follows: perception on how easy it is to contract HPV, level of trust in the medical profession as a source of information on prevention, overall perception on the importance of children/adolescents' vaccination, physician as a source of information about the virus, whether the daughter's treating physician has recommended HPV vaccination, internet/social media as a source for HPV information, age of the participant parent and degree of avocation with religion. Initial model construction analysis including sociodemographic variables failed to detect any statistically significant associations with the dependent variable, thus, most sociodemographic characteristics were not used in the final model analysis, which was performed with the Enter method.

## Results

### Sample Characteristics

Overall, the study sample consisted of 1,000 parents of girls aged 11–18 years old at the time of the survey. The main characteristics of the sample are presented in Table 1. Almost all participants were female (99.0%, *n* = 990), with a mean age of 45.56 years old (SD = 5.005), while the majority was married/living with a spouse (88.1%, *n* = 879). Regarding education, 42.5% (*n* = 421) of the study sample had tertiary education and more than 6 out of 10 participants were occupied as employees (67.8%, *n* = 671). Also, almost every participant had public social insurance (97.6%, *n* = 951). Additionally, the participants were asked to state the level of their avocation with religion, resulting in a mean score of 6.98 (SD = 2.134). The medical profession was found to be the most trusted source of information on health prevention issues (mean score = 7.91, SD = 1.542, *n* = 973) when compared to other sources, like the Ministry of Health, the National Drug Organization, pharmacists, the pharmaceutical industry, the internet, and the press.

**Table 1:** Sociodemographic characteristics of the study sample.

	**Frequency (%)**
**Gender**	
Male	10 (1.0)
Female	990 (99.0)
**Family status**	
Married/living with spouse	879 (88.1)
Divorced/separated	110 (11.0)
Widowed	7 (0.7)
Not married, living with relatives	2 (0.2)
**Educational status**	
Elementary	13 (1.3)
Secondary/high school	556 (56.2)
Tertiary	421 (42.5)
**Occupational status**	
Employer/self-employed	147 (14.9)
Employee	671 (67.8)
Unemployed	39 (3.9)
Retired	14 (1.4)
Household	116 (11.7)
Other	2 (0.2)
**Monthly family income**	
No income	0 (0.0)
1–500€	8 (1.5)
501–1,000€	84 (15.4)
1,001–1,500€	134 (24.5)
1,501–2,000€	181 (33.2)
2,001–3,000€	115 (21.1)
3,001+€	24 (4.4)
**Social insurance (public)**	
Yes	951 (97.6)
No	23 (2.4)

### Results Regarding HPV

According to the analysis, 99.4% (*n* = 992) of the study sample knew about Human Papilloma Virus and the main source of information regarding the virus were physicians (91.5%, *n* = 911), internet/social media (45.5%, *n* = 453), and friends/family (43.7%, *n* = 435). Intercourse was perceived as the main mode of HPV transmission by the majority of participants (95.6%, *n* = 917), while a small proportion thinks that HPV can be transmitted with common use of syringes (14.6%, *n* = 140). Regarding prevention of HPV transmission, use of condom during intercourse was the first choice for 87.6% (*n* = 842) of the study sample, while the second most common choice was Pap smear (35.0%, *n* = 336).

Concerning the perceptions of the study participants concerning which diseases are associated with HPV, the majority recognizes that it is linked with cervical cancer (89.0%, *n* = 879) and genital and anal warts (73.5%, *n* = 726). Also, the participants were asked how cervical cancer can be prevented, with 3 out of 4 (75.8%, *n* = 750) choosing HPV vaccination as the main preventive method.

Furthermore, participants were asked to rate the seriousness of the consequences associated with contracting HPV on a 0–10 scale (0: not at all serious; 10: extremely serious), which derived a mean score of 8.77 (SD = 1.241, *n* = 974), while they were also asked to assess how easy it is to get the virus, which resulted in a mean score of 7.50 (SD = 2.027, *n* = 964).

### Attitudes and Perceptions Toward HPV Vaccine

Overall, 98.8% (*n* = 986) of the study participants know that there is a vaccine against HPV, with health care professionals being the most common source of information regarding the vaccine (88.7%, *n* = 875). The effectiveness of HPV vaccine is recognized by the participants, since the majority of them consider it high and very high in prevention of genital warts (74.1%, *n* = 598) and cervical cancer (77.0%, *n* = 638). Furthermore, the role of their daughters' treating physician concerning HPV vaccination was investigated. Based on the answers of the respondents, more than 9 out of 10 physicians have informed the parents/daughters about HPV vaccination (92.7%, *n* = 836) and have recommended it (92.1%, *n* = 758).

As far as actual vaccination against HPV is concerned, almost half of the participants stated that their daughters had been vaccinated (47.0%, *n* = 468). However, further analysis on the number of doses the girls had received, revealed that only 35.0% (*n* = 345) of the overall sample was fully vaccinated up until the survey (2 doses for girls vaccinated at age <15y.o. and 3 doses for girls vaccinated at age ≥15y.o.). The main reason that drove their decision to have their daughters vaccinated was physician recommendation (50.8%, *n* = 235), while an equally significant proportion of participants stated that they wanted to protect their daughters from being infected with HPV in the future (47.3%, *n* = 219) (Table 2).

**Table 2:** Reasons for daughter's vaccination status.

**Participants whose daughter has been vaccinated**	
**Main reason for vaccination decision**	***n*** **(%)**
Physician recommendation	235 (50.8)
I wanted to protect my daughter from being infected with HPV in the future	219 (47.3)
Person in close environment with cervical cancer	9 (1.9)
**Participants whose daughter has not been vaccinated**	
**Reasons for not vaccinating** ^1^	***n*** **(%)**
I do not have enough information available to decide	214 (41.4)
My daughter is too young to be vaccinated against HPV	187 (36.2)
The vaccine is very new	118 (22.8)
I do not believe that the vaccination is safe	109 (21.1)
My daughter does not have sexual activity	82 (15.9)
It was not recommended by the treating physician	52 (10.1)
**Do you plan to vaccinate your daughter in the future?**	
Yes	227 (68.2)
**Planning for future vaccination**	
Within the next 6 months	88 (42.5)
Within the next 6 months−1 year	45 (21.7)
More than 1 year in the future	74 (35.7)

1 *Question with multiple responses*.

On the other hand, regarding those participants who had not had their daughters vaccinated up to the time of the survey, the most common reasons were that they did not have enough information available to decide (40.5%, *n* = 214) and that their daughters were too young to be vaccinated against HPV (35.4%, *n* = 187). It should be noted than 1 in 5 participants (20.7%, *n* = 108) stated that they did not believe that the vaccine is safe as a reason for not vaccinating their daughters. Nevertheless, 68.2% (*n* = 227) of those who had not vaccinated their daughters declared that they plan to do it in the future. Finally, the participants were asked to state their overall agreement/disagreement toward importance of vaccination in general for children, which resulted in agreement from more than 9 out of 10 participants (91.0%, *n* = 863).

### Factors Associated With HPV Vaccination

Based on the univariate analyses, marital status, occupation, education, income, and public social insurance coverage of the study sample were not found to be associated with vaccination status of the sample's daughter. Similarly, degree of avocation with religion did not differ among the parents who had and those who had not vaccinated their daughter. Parents reporting vaccinated daughter were somewhat, but at statistically significant level, older (*p* < 0.001) (mean age = 46.64 years, SD = 4.548, *n* = 468) than their counterparts not having vaccinated their daughter (mean age = 44.59 years, SD = 5.211, *n* = 528). Trust in the medical profession was higher (mean score: 8.21, SD = 1.453, *n* = 461) among respondents who reported having vaccinated their daughter than their counterparts not having vaccinated their daughter (mean score = 7.64, SD = 1.565, *n* = 508) (*p* < 0.001).

Perceived ease that somebody contracts HPV during his/her lifetime was found to be significantly higher (*p* < 0.001) for respondents who had vaccinated their daughter (mean score = 7.8, SD = 1.946, *n* = 457) when compared to those who had not done so (mean score = 7.21, SD = 2.059, *n* = 503).

Among parents reporting vaccination coverage of their daughter aged 11–18 years, 467 (99.8%) had knowledge of the HPV virus, as expected, while 450 (96.2%) reported physician as their source of information about the virus, this being by far the most important and positively associated factor with vaccination coverage (Table 3). In contrast, negative impact on vaccination coverage seem to have the internet/social media and friends/family as sources of information about the HPV virus. Although the vast majority (95.9%) acknowledges that sexual intercourse is the means for HPV transmission, this knowledge is not associated with vaccination coverage, while also the knowledge of the majority (87.8%) that the use of condom during intercourse may prevent HPV is not associated with vaccination coverage. Additionally, knowledge that cervical cancer of the uterus (93.1%) is associated with the HPV virus and that vaccination against HPV offers prevention (82.5%) are significantly related to vaccination coverage.

**Table 3:** Parents' knowledge and sources of information about the HPV virus, and univariate associations with vaccination coverage of their daughter aged 11–18 years (incl. Risk Ratio and 95% C.I.).

	**HPV vaccination coverage**		
	**Vaccinated *n* (%)**	**RR**	**95% CI of RR**
**Knowledge of HPV as a virus**			
Yes	467 (99.8)	2.84	0.47–16.99
**Source of information on HPV** ^1^			
Physician^***^	450 (96.2)	2.28	1.51–3.45
Internet/social media^***^	186 (39.7)	0.79	0.69–0.91
Friends/family^*^	185 (39.5)	0.85	0.74–0.97
Television/radio	151 (32.3)	0.89	0.77–1.03
Pharmacist	59 (12.6)	0.91	0.74–1.11
Other	3 (0.6)	1.06	0.48–2.37
**Perceptions on means of HPV transmission** ^1^			
Intercourse	439 (95.9)	1.06	0.76–1.49
Other sexual activities	177 (38.6)	1.11	0.97–1.27
Shared use of syringes among individuals	74 (16.2)	1.13	0.95–1.34
Labor (transmission from mother to child)	37 (8.1)	1.17	0.93–1.46
Skin contact	29 (6.3)	1.09	0.84–1.41
Pregnancy^*^	17 (3.7)	1.56	1.21–2.01
**Perceptions regarding prevention of HPV transmission** ^1^			
Use of condom during intercourse	403 (87.8)	1.02	0.83–1.25
Pap smear	157 (34.2)	0.97	0.84–1.11
Delayed initiation of sexual activity	121 (26.4)	1.12	0.97–1.29
Use of contraceptive pills	38 (8.3)	1.06	0.84–1.34
Other^**^	58 (12.6)	1.31	1.10–1.57
**Perceptions regarding diseases associated with HPV** ^1^			
Cervical cancer^***^	430 (93.1)	1.67	1.24–2.26
Genital & anal warts	352 (76.2)	1.14	0.97–1.34
Precancerous lesions in the cervix	229 (49.6)	1.05	0.92–1.20
Genital & anal cancer	184 (39.8)	1.01	0.88–1.16
Oropharyngeal cancer	81 (17.5)	1.13	0.95–1.34
**Perceptions regarding cervical cancer prevention** ^1^			
Vaccination against HPV^***^	386 (82.5)	1.50	1.24–1.81
Use of condom during intercourse	292 (62.4)	0.94	0.82–1.08
Pap smear	250 (53.4)	1.05	0.92–1.19
Delayed initiation of sexual activity	92 (19.7)	1.09	0.93–1.28
Use of contraceptive pills	32 (6.8)	0.99	0.76–1.29

1 *Question with multiple responses*,

* *p < 0.05*,

** *p < 0.01*,

*** *p < 0.001 (chi-square)*.

Overall, HPV vaccination coverage of daughters aged 11–18 year is significantly related (Table 4) to their parents' having been informed by a healthcare professional about the existence of the HPV vaccine, having received recommendation by their daughter's treating physician, perceiving as high the effectiveness of the HPV vaccine in preventing both genital warts and cervical cancer of the uterus and having stronger belief on the importance of children's vaccinations in general.

**Table 4:** Parents' knowledge and attitudes about HPV vaccination, and univariate associations with vaccination coverage of their daughter aged 11–18 years (incl. Risk Ratio and 95% C.I.).

	**HPV vaccination coverage**		
	**Vaccinated *n* (%)**	**RR**	**95% CI of RR**
**Knowledge of the existence of HPV vaccine**			
Yes	466 (99.6)	2.85	(0.80–10.11)
			
**Source of information on HPV vaccine** ^1^			
Health care professional^***^	431 (92.1)	1.64	(1.24–2.17)
Internet	214 (45.7)	0.98	(0.86–1.12)
Pharmacist	75 (16.0)	0.90	(0.75–1.09)
Friends/family	194 (41.5)	0.92	(0.80–1.05)
Television/radio	155 (33.1)	0.98	(0.85–1.13)
Printed material (leaflets, newspapers, magazines etc.)	63 (13.5)	1.16	(0.96–1.39)
Woman or man in the friend/family environment with warts/cervical cancer/other types of cancer	21 (4.5)	0.93	(0.67–1.29)
Informed by the daughter's treating physician about the HPV vaccination^***^			
Yes	414 (98.1)	3.80	(1.98–7.28)
**Recommendation for HPV vaccination by the daughter**'s treating physician^***^			
Yes	406 (98.1)	4.37	(2.28–8.40)
Perceived effectiveness of HPV vaccine on genital warts prevention^***^			
Low	7 (1.7)		
Moderate	47 (11.5)		
High	236 (57.6)		
Very high	120 (29.3)		
Perceived effectiveness of HPV vaccine on cervical cancer prevention^***^			
Low	10 (2.4)		
Moderate	38 (9.0)		
High	245 (57.8)		
Very high	131 (30.9)		
Overall, I think vaccines are important for children to have^***^			
Strongly agree	317 (70.4)		
Agree	112 (24.9)		
Disagree	16 (3.6)		
Strongly disagree	5 (1.1)		

1 *Question with multiple responses*,

*** *p < 0.001 (chi-square)*.

The results of the binary regression model are presented in Table 5. Statistically significant association with HPV vaccination was found for the following variables: perceived ease of contracting HPV during one's lifetime, level of trust in medical profession to provide information on prevention, overall perception regarding importance of children's vaccination, internet/social media as a source of information regarding HPV, participant's age and daughter's treating physician's recommendation for HPV vaccination.

**Table 5:** Factors associated with (reported) HPV vaccination.

	***p*-value**	**Odds Ratio**	**Std. Error**	**95% CI**	
				**Lower**	**Upper**
Perceived ease of contracting HPV during one's lifetime	0.016	1.105	0.041	1.018	1.198
Level of trust in medical profession regarding information on prevention	0.001	1.205	0.055	1.081	1.343
Overall perception regarding importance of children's vaccination	0.000	0.618	0.116	0.493	0.776
Physician as a source of parent information on HPV	0.103	1.931	0.404	0.876	4.258
Internet/social media as a source of parent information on HPV	0.023	0.886	0.053	0.695	1.143
Participant (parent) age	0.000	1.125	0.017	1.087	1.164
Daughter's treating physician's recommendation for her HPV vaccination	0.000	7.319	0.406	3.305	16.209
Degree of avocation with religion	0.742	0.987	0.039	0.914	1.066
Constant	0.000	0.000	1.203		

Specifically, probability of HPV vaccination increases as the perceived ease of contracting HPV increases (OR = 1.105; 95% CI = 1.018, 1.198), as level of trust in medical profession to provide information on prevention increases (OR = 1.205; 95% CI = 1.081, 1.343) and as the participant age increases (OR = 1.125; 95% CI = 1.087, 1.164). Furthermore, as the perceived importance of children's vaccination decreases, the probability of HPV vaccination decreases (OR = 0.618; 95% CI = 0.493, 0.776). Also, a particularly strong positive statistical association was found between daughter's treating physician's recommendation for HPV vaccination and HPV vaccination (OR = 7.319; 95% CI = 3.305, 16.209). On the other hand, probability of HPV vaccination is lower for those who have internet/social media as a source of information regarding HPV, compared to persons who have other sources of information on the virus (OR = 0.886; 95% CI = 0.695, 1.143).

No statistically significant association with HPV vaccination was found for the variables “Physician as a source of parent information on HPV” and “degree of avocation with religion.”

## Discussion

HPV is a serious public health issue, as it is responsible for the overall incidence of cervical cancer, while it is also related to various other types of cancer. The development of an effective and safe HPV vaccine has been an important achievement. However, wide acceptance of the vaccine by the public is yet to be achieved, despite the increasing literature evidence supporting its effectiveness and safety. The present study is the first extensive and multifactorial investigation of HPV vaccination coverage in Greece, as well as of the determinants associated with vaccination.

According to the results of the analysis, HPV is common knowledge among Greek parents of adolescent girls, with medical community emerging as the main source of information. On the other hand, a study on Greek adolescents' knowledge on HPV has revealed that only 43% of them know about the virus (17), which indicates that education on HPV should improve and be integrated in younger ages. Regarding perceptions on HPV transmission, the majority of the participants correctly know that sexual intercourse is the main mode of transmission, while they also correctly know that use of condom is the main preventive method against HPV transmission, findings that are both in line with previous relevant studies (17, 18). However, it should be noted that a sizeable proportion of the sample (1 out of 3) have the wrong perception that Pap smear is the main preventive method against HPV transmission, while also a small proportion falsely thinks that HPV can be transmitted with common use of syringes. Furthermore, knowledge regarding the diseases that HPV is associated with can be considered adequate, as the vast majority are aware that it is associated with cervical cancer and genital and anal warts, while in contrast, other recent studies in the US and Italy have showed that, while most people are aware that HPV is sexually transmitted, many do not know that it can cause cervical cancer and even less that it is linked with genital warts (18, 19). Based on the above, the fact that the majority of participants have knowledge of HPV and its implications highlights the improvement in raising awareness during the last decade in Greece, compared to relevant previous international research (20, 21), while relevant studies in other countries show lower awareness, such as Poland, where only 62.5% of the participants had ever heard of HPV (22).

Almost all study participants know the existence of the HPV vaccine, with healthcare professionals and more particularly pediatricians, pathologists, and gynecologists as their main source of information. Additionally, the effectiveness of HPV vaccine is widely recognized for both the prevention of genital warts and cervical cancer, which is in line with the international literature (19). Regarding HPV vaccination practices, the first impression from the results of the analysis is that, according to the participating parents, almost half of the girls have been vaccinated against HPV. However, further analysis of the data on the basis of the required doses of the vaccine according to the national guidelines, revealed that only 35.0% of the girls are fully vaccinated against HPV. This finding is in line with another recent Greek study where 39.3% of women had received the HPV vaccine, although this study did not involve girls aged 11–18, but a broader age group of 12–26 years old (23). For those whose daughters have not received any dose of the HPV vaccine, the main reason was that they do not have enough information to make such decision and that their daughter is too young to be vaccinated. Accordingly, a relevant research study in the US found that parents of adolescents not vaccinated against HPV who were very or somewhat hesitant to proceed to vaccination chose safety concerns/side effects as the main reason, while those unsure chose lack of provider recommendation and lack of knowledge as the main reasons (24).

The role of healthcare professionals in the HPV vaccination is highlighted, since half the participants stated that physician recommendation was the main reason for their decision to vaccinate their daughters against HPV. Additionally, according to the results of the binary logistic regression analysis, physician recommendation is the factor with the highest positive association with the decision to vaccinate one's daughter against HPV. Physician influence has been consistently observed in the international literature, as recommendation for HPV vaccination is associated with higher acceptance rates (25, 26). Moreover, a previous study has determined that recommendation for HPV vaccination is not only a matter of quantity but also of quality, since parents with high-quality recommendation were significantly more likely to proceed to initiation and follow-through of all the doses of the vaccine, while they were also less likely to refuse or delay HPV vaccination (25).

Overall, childhood vaccination rates in Greece are remarkably high, exceeding 95% (27), which is indicative of the commitment of the public and the state in such preventive measures, although there is no recent official record regarding HPV vaccination coverage. Additionally, the study results show that public agreement with importance of children's vaccination is high, which is in overall alignment with the respective results of the Vaccine Confidence Project, the purpose of which is to monitor public confidence in immunization programmes (28).

Given that HPV vaccination has optimal effectiveness when performed in early adolescence (11 years old), while in Greece it is also available for girls aged 9 years old, special consideration should be given in order to enhance provision of valid information to the wider public and especially parents regarding this recommendation and its justification, based on available research data. More importantly, this information should be conveyed in a simple and comprehensive way, in order to be understood by the general population.

Based on the findings, the present study confirms that optimal coverage of HPV vaccination is yet to be achieved. The role of healthcare professionals has been highlighted as a significant factor in the parents' decision to vaccinate their daughters against HPV, which can and should be utilized in order to increase public acceptance of the vaccine. Further research on the reasons why parents decide to not vaccinate their daughters against HPV would be of great interest in the field.

### Study Strengths and Limitations

The size of the study sample and the fact that it is representative of the overall population of parents of girls aged 11–18 years old in Greece, based on the methodology and design of the study, constitute an important advantage for the validity of the results of the analysis.

However, the present study may be subject to a few limitations, as is common in similar research studies. As the objective of the study involves a serious health issue and the participants' responses may reflect their “interest” in their children's health, the possibility of social desirability bias cannot be excluded. Also, a number of questions refer to past time (i.e., age of vaccination and number of doses received) and therefore recall bias may be present. Furthermore, adolescents, who are the main interested party in the study objective, were not included as participants in the survey.

## Data Availability Statement

The raw data supporting the conclusions of this article will be made available by the authors, without undue reservation.

## Ethics Statement

The studies involving human participants were reviewed and approved by Bioethics and Research Ethics Committee of the Hellenic National School of Public Health (3568/9.11.2018). Written informed consent for participation was not required for this study in accordance with the national legislation and the institutional requirements.

## Author Contributions

KA, JK, and EP: conceptualization and supervision. KA and EP: methodology. EP and PN: design of research tools. PN, DZ, and KA: formal analysis and investigation. PN: writing—original draft preparation. KA, DZ, JK, and EP: writing—review and editing. All authors contributed to the article and approved the submitted version.

## Funding

The present study was financially supported by MSD Hellas.

## Conflict of Interest

The authors declare that the research was conducted in the absence of any commercial or financial relationships that could be construed as a potential conflict of interest.

## Publisher's Note

All claims expressed in this article are solely those of the authors and do not necessarily represent those of their affiliated organizations, or those of the publisher, the editors and the reviewers. Any product that may be evaluated in this article, or claim that may be made by its manufacturer, is not guaranteed or endorsed by the publisher.
